# Remimazolam Versus Propofol for General Anesthesia in Older Adults Undergoing Colon Cancer Surgery: A Systematic Review and Meta-Analysis of Comparative Studies

**DOI:** 10.3390/pharmaceutics18040448

**Published:** 2026-04-06

**Authors:** Khalid I. AlHussaini, Ibrahim Abdullah Abalhassan, Eman Toraih, Abdullah Ibrahim Alhussaini

**Affiliations:** 1 Department of Internal Medicine, College of Medicine, Imam Mohammad Ibn Saud Islamic University (IMSIU), Riyadh 11564, Saudi Arabia; 2 Department of Anesthesia, King Abdulaziz Medical City, Ministry of National Guard—Health Affairs, Riyadh 11564, Saudi Arabia; ibabalhassan@gmail.com (I.A.A.); dralhussaini95@gmail.com (A.I.A.); 3 Department of Cardiovascular Perfusion, College of Health Professions, Upstate Medical University, Syracuse, NY 13210, USA; toraihe@upstate.edu

**Keywords:** remimazolam, propofol, colon cancer surgery, colorectal cancer surgery

## Abstract

**Background:** Propofol is widely used for anesthesia in colorectal cancer surgery, but is frequently associated with hypotension and respiratory depression. Remimazolam, a novel ultra-short–acting benzodiazepine, may offer improved hemodynamic stability with similar anesthetic depth and recovery characteristics. However, evidence directly comparing remimazolam and propofol in the setting of colon cancer surgery remains limited. Therefore, the aim of this study was to systematically evaluate the efficacy, safety, perioperative hemodynamic stability, and recovery outcomes of remimazolam versus propofol in older adults undergoing colon cancer surgery. **Methods:** We conducted a systematic review and meta-analysis of randomized controlled trials and comparative cohort studies evaluating remimazolam versus propofol in adult patients undergoing colon or colorectal cancer surgery. PubMed, Scopus, and Web of Science were searched from the start of each database to October 2025. Outcomes included perioperative hemodynamics (MAP and HR), recovery parameters, intraoperative remifentanil consumption, anesthesia duration, and adverse events. Random-effect models were used to calculate pooled mean differences (MDs) or risk ratios (RRs) with 95% confidence intervals (CIs). **Results:** Six studies involving 542 patients (remimazolam *n* = 276; propofol *n* = 266) were included. Remimazolam produced significantly higher perioperative MAP (overall MD = 2.86 mmHg, 95% CI 1.52–4.21; *p* < 0.0001) and slightly higher HR (MD = 2.30 bpm, 0.08–4.52; *p* = 0.04). Differences were largest immediately after incision and at the end of surgery. No significant differences were found in PACU stay, overall recovery duration, remifentanil consumption, or anesthesia duration. Postoperative nausea and vomiting were comparable (RR = 0.93; *p* = 0.86), while respiratory depression was numerically lower with remimazolam (RR = 0.49; *p* = 0.17). **Conclusions:** Remimazolam provides anesthetic efficacy comparable to propofol in colon cancer surgery while offering modest, but clinically meaningful improvements in intraoperative hemodynamic stability. Recovery times, opioid requirements, and adverse-event rates were similar between agents. Remimazolam may be particularly advantageous for elderly or hemodynamically vulnerable patients undergoing major colorectal procedures. Larger, high-quality trials are warranted to clarify long-term and oncologic outcomes.

## 1. Introduction

Colorectal cancer ranks third in incidence and second in mortality among all malignant tumors, and its burden has continued to rise in recent years [1,2,3]. Surgical resection remains the primary therapeutic approach; however, both surgery and anesthesia can activate the hypothalamic–pituitary–adrenal axis and the sympathetic nervous system, resulting in immunosuppression that may facilitate tumor micrometastasis and increase the risk of postoperative recurrence [4,5,6].

Propofol primarily acts as a positive allosteric modulator and direct agonist of the GABA-A receptor, enhancing inhibitory neurotransmission. It also inhibits NMDA receptors and modulates calcium influx, contributing to its sedative and cardiovascular depressant effects. It offers the advantages of rapid onset and quick recovery, making it a commonly used anesthetic in gastroscopy [7]. However, its administration can rapidly induce adverse effects, including hypotension and hypoxemia [8,9,10], and injection-related pain remains a notable concern [11].

Remimazolam is a novel short-acting intravenous benzodiazepine that functions as a positive allosteric modulator of the γ-aminobutyric acid subtype A (GABA_A) receptor through the benzodiazepine binding site [12,13]. It retains the classical features of benzodiazepines, including water solubility, reversibility with antagonists, and absence of injection pain. In addition, due to the presence of a methyl propionate side chain in its chemical structure, remimazolam exhibits pharmacological properties similar to remifentanil, such as rapid onset, short duration of action, metabolite inactivity, and pharmacokinetics that are not significantly affected by infusion duration [14,15,16]. Studies have reported that the sedation success rate of remimazolam is non-inferior to that of propofol while being associated with a lower incidence of adverse events [17,18].

Despite growing clinical use, the optimal sedation regimen for colorectal cancer surgery remains unclear. Specifically, evidence comparing propofol and remimazolam in terms of sedation stability, safety, recovery profile, and procedural efficiency is limited. This study aimed to evaluate the effectiveness, safety, and procedural stability of remimazolam vs. propofol in older adults undergoing colorectal cancer surgeries.

## 2. Materials and Methods

This systematic review was designed in accordance with the Preferred Reporting Items for Systematic Reviews and Meta-Analyses (PRISMA) 2020 guidelines (Table S1) [19].

### 2.1. Searching and Screening

A comprehensive literature search was conducted to identify comparative studies evaluating remimazolam versus propofol in adults undergoing colon cancer surgery under general anesthesia. We systematically searched PubMed, Scopus, and Web of Science from the start of each database to October 2025, using combinations of keywords and MeSH terms related to “remimazolam” AND “propofol” AND “colon cancer” OR “colorectal cancer.” The search strategy was adapted for each database, and reference lists of relevant studies and reviews were also screened to ensure completeness. All retrieved records were imported into a reference management program, and duplicates were removed. Titles and abstracts were screened first, followed by full-text assessments of potentially eligible articles.

### 2.2. Eligibility Criteria

Studies were eligible if they were randomized controlled trials (RCTs) or comparative cohort studies involving adult patients undergoing elective colon or colorectal cancer surgery under general anesthesia, directly comparing remimazolam with propofol, and reporting at least one relevant clinical outcome. These outcomes included hemodynamic parameters such as mean arterial pressure (MAP) and heart rate (HR) at predefined perioperative timepoints, recovery metrics including post-anesthesia care unit (PACU) stay and time to emergence, intraoperative variables such as remifentanil consumption and anesthesia duration, and safety outcomes including postoperative nausea and vomiting or respiratory depression. Studies were excluded if they were non-comparative, conducted in non-surgical or pediatric populations, involved sedation outside colorectal surgery, or lacked extractable quantitative data. Reviews, case reports, and conference abstracts were excluded as well.

### 2.3. Data Extraction

Data extraction was performed using a standardized form. Extracted information included study characteristics, patient demographics such as age, gender, and body mass index (BMI), surgical and anesthetic details including surgical duration, and American Society of Anesthesiologists (ASA) class, hemodynamic measurements at defined intraoperative and postoperative timepoints, recovery outcomes, intraoperative analgesic use, anesthesia duration, and reported adverse events.

### 2.4. Quality and Risk-of-Bias Assessment

RCTs were evaluated using the Cochrane Risk of Bias 2.0 (Rob-2) tool, assessing bias in randomization, deviations from intended interventions, missing outcome data, measurement of outcomes, and selective reporting [20]. Studies were rated as having low, some concerns, or high risk of bias. Non-randomized comparative studies were assessed using the Newcastle–Ottawa Scale (NOS), evaluating selection, comparability, and outcome domains [21]. Studies were categorized as low (0–3 stars), moderate (4–6 stars), or high quality (7–9 stars).

### 2.5. Statistical Analysis

We conducted meta-analyses using a random-effect model for outcomes that were pooled from at least three studies. We summarized continuous outcomes using mean difference (MD) with 95% confidence intervals (CIs), and binary outcomes as risk ratios (RRs) with 95% CIs. We assessed between-study heterogeneity using the I^2^ statistic and the *p*-value from the chi-squared test, considering heterogeneity significant when I^2^ > 50% and *p* < 0.1. We generated forest plots to visualize the effect sizes of individual studies as well as the overall pooled effect. We conducted a subgroup analysis based on time for the hemodynamic outcomes. For all analyses, we considered statistical significance at *p* < 0.05. We performed all statistical analyses using RevMan 5.4 software.

## 3. Results

**Figure 1:**
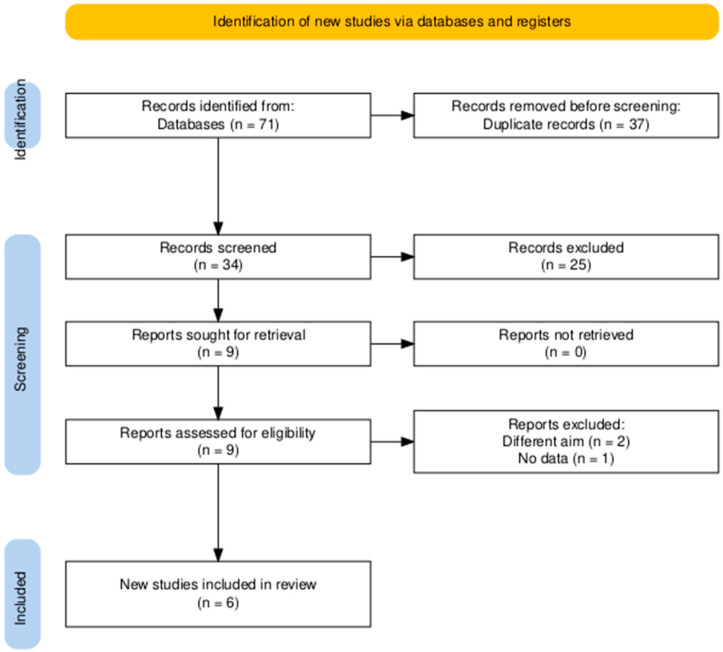
PRISMA flow diagram of searching and screening.

The search strategy resulted in a total of 71 articles, including 37 duplicates. We conducted title and abstract screening on the remaining 34 articles. Full-text screening was undertaken on 9 articles after exclusion of 25 articles in the previous step. Ultimately, we included six articles in the current systematic review and meta-analysis [22,23,24,25,26,27] (Figure 1).

### 3.1. Characteristics of Studies

Six comparative studies (five RCTs and one cohort study) were included, enrolling a total of 542 participants (remimazolam, *n* = 276; propofol, *n* = 266). All studies involved older adults undergoing elective colon cancer surgery under general anesthesia. Baseline characteristics were closely matched between groups. Mean age across trials ranged from 54 to 74 years (overall average = 64 years), with males comprising roughly half of participants (44%–71%). Body mass index clustered in the low to mid-20s and was similar by arm (means 21.6–24.2 kg/m^2^; pooled average ~22.7 kg/m^2^). Where available, most trials reported anesthesia/operative times around 190–236 min, with similar means between arms. ASA physical status was predominantly I–II and well balanced (mean proportions = 51% ASA I and 47% ASA II across arms). Detailed baseline characteristics are shown in Table 1.

### 3.2. Quality and Risk-of-Bias Assessment

**Figure 2:**
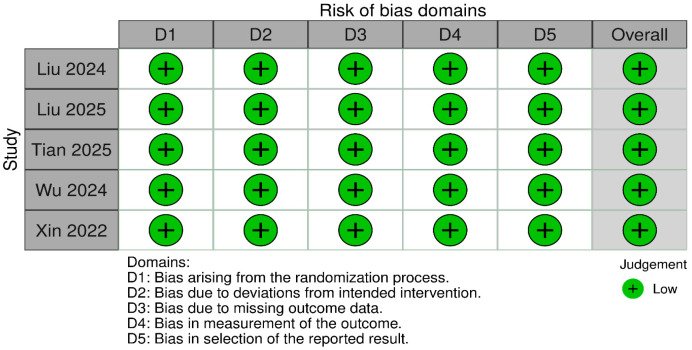
Risk-of-bias assessment of RCTs using Rob-2 [22,23,25,26,27].

According to Rob-2, the five RCTs had a low risk of bias (Figure 2), and according to NOS, the cohort study had high quality (Table 2).

### 3.3. Hemodynamic Outcomes

**Figure 3:**
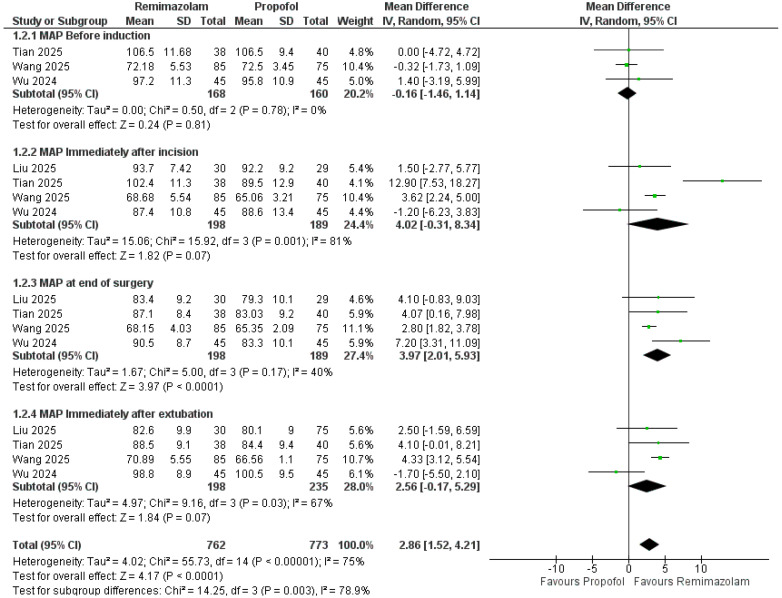
Mean arterial pressure (MAP) with remimazolam vs. propofol at perioperative timepoints [23,24,25,26].

**Figure 4:**
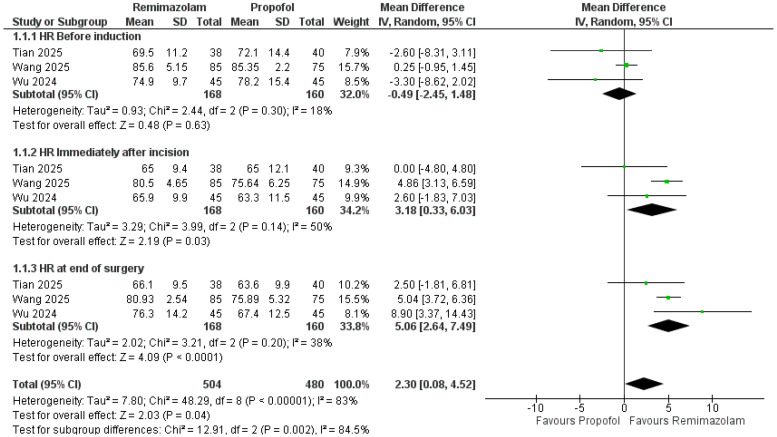
Heart rate (HR) with remimazolam vs. propofol at perioperative timepoints [24,25,26].

Across timepoints, remimazolam was associated with higher MAP versus propofol (Figure 3). Pooled over all four timepoints, MAP was higher by 2.86 mmHg (95% CI 1.52–4.21; *p* < 0.0001; I^2^ = 75%). By subgroup, there was no difference before induction (MD = −0.16 mmHg, 95% CI −1.46 to 1.14; I^2^ = 0%; *p* = 0.81), a non-significant trend toward higher MAP immediately after incision (MD = 4.02 mmHg, −0.31 to 8.34; I^2^ = 81%; *p* = 0.07), a significant increase at the end of surgery (MD = 3.97 mmHg, 2.01 to 5.93; I^2^ = 40%; *p* < 0.0001), and a non-significant trend immediately after extubation (MD = 2.56 mmHg, −0.17 to 5.29; I^2^ = 67%; *p* = 0.07). HR was slightly higher overall with remimazolam (MD = 2.30 bpm, 0.08–4.52; *p* = 0.04; I^2^ = 83%), with no difference before induction (MD = −0.49 bpm, −2.45 to 1.48; I^2^ = 18%; *p* = 0.63), but higher immediately after incision (MD = 3.18 bpm, 0.33–6.03; I^2^ = 50%; *p* = 0.03) and at the end of surgery (MD = 5.06 bpm, 2.64–7.49; I^2^ = 38%; *p* < 0.0001) (Figure 4).

### 3.4. Perioperative Outcomes

**Figure 5:**
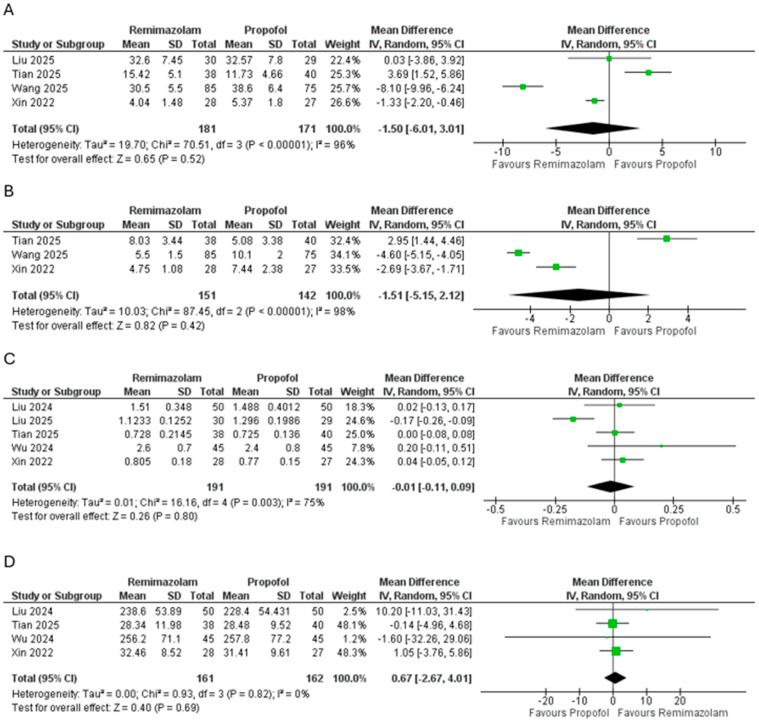
Pooled effect size of (**A**) length of stay in PACU (min), (**B**) recovery duration, (**C**) remifentanil dosage (mg), and (**D**) anesthesia duration (min) between the groups [22,23,24,25,26,27].

There were no significant between-group differences in recovery or operative metrics (Figure 5). Length of stay in the PACU did not differ (MD = −1.50 min, 95% CI −6.01 to 3.01; *p* = 0.52; I^2^ = 96%), nor did overall recovery duration (MD = −1.51 min, −5.15 to 2.12; *p* = 0.42; I^2^ = 98%). Intraoperative remifentanil consumption was comparable (MD = −0.01 mg, −0.11 to 0.09; *p* = 0.80; I^2^ = 75%), and total anesthesia duration showed no difference (MD = 0.67 min, −2.67 to 4.01; *p* = 0.69; I^2^ = 0%).

### 3.5. Safety Outcomes

**Figure 6:**
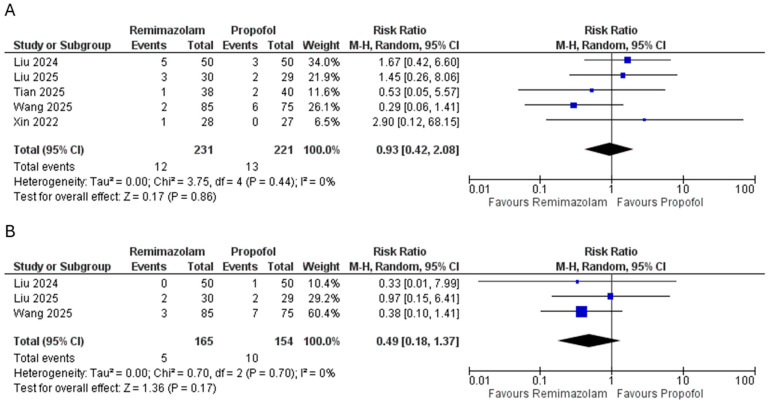
Pooled effect size of (**A**) nausea and vomiting and (**B**) respiratory depression between the groups [22,23,24,26,27].

Postoperative nausea and vomiting were similar between the remimazolam and propofol groups (RR = 0.93, 95% CI 0.42–2.08; *p* = 0.86; I^2^ = 0%) (Figure 6A). Respiratory depression was numerically less frequent with remimazolam, but not statistically significant (RR = 0.49, 95% CI 0.18–1.37; *p* = 0.17; I^2^ = 0%) (Figure 6B).

## 4. Discussion

### 4.1. Summary of Findings

Our meta-analysis of six trials found that remimazolam and propofol provided similar anesthetic efficacy in colon cancer surgery, but with modest hemodynamic differences favoring remimazolam. Pooled data showed higher MAP with remimazolam. Subgroup analyses indicated larger MAP increases later in surgery and smaller non-significant trends immediately post-incision or post-extubation. HR was also slightly higher with remimazolam, driven by differences immediately after incision and at surgery end. While remimazolam was associated with statistically higher MAP and HR values, the absolute differences were modest. A mean MAP difference of approximately 3 mmHg and HR difference of 2 bpm are unlikely to translate into major clinical impact in otherwise stable patients. Therefore, the statistical significance observed should not be interpreted as large hemodynamic superiority. In contrast, there were no significant differences in recovery or operative metrics: PACU stay, overall recovery time, intraoperative remifentanil use, or total anesthesia duration. Adverse-event rates were comparable: postoperative nausea/vomiting was essentially identical, while respiratory depression trended lower with remimazolam, but without statistical significance. Thus, remimazolam conferred significantly higher MAP and slightly higher HR during surgery, without any trade-off in recovery speed or opioid requirement. These differences, although numerically small, likely translate to fewer episodes of hypotension and less need for vasopressors (as seen in related trials), while providing equivalent anesthetic recovery.

### 4.2. Hemodynamic Stability

Remimazolam demonstrated superior hemodynamic stability compared to propofol. Pharmacologically, propofol causes marked systemic vasodilation and myocardial depression (via GABA_A and NMDA pathways), whereas remimazolam (a benzodiazepine) exerts milder cardiovascular effects [28,29]. Our pooled analysis consistently shows this: remimazolam maintained higher blood pressure during and after induction. He et al. [30] found that remimazolam preserved systemic vascular resistance (SVR) and blunted the MAP drop seen with propofol. Clinically, Gu et al. [31] reported significantly fewer hypotension and bradycardia events in elderly patients sedated with remimazolam (37% vs. 73% hypotension, *p* < 0.001; 7% vs. 16% bradycardia, *p* = 0.037). In line with those findings, a recent meta-analysis in older adults showed remimazolam roughly halved the incidence of intraoperative hypotension (RR = 0.5) and tended to reduce bradycardia (RR = 0.56) [29].

These differences take on special significance in elderly surgical patients. Aging causes decreased β-receptor responsiveness, vascular stiffness, and reliance on adequate preload and atrial filling for cardiac output. In other words, geriatric hearts depend heavily on maintaining end-diastolic volume and cannot compensate well for drops in blood pressure [32]. Even mild intraoperative hypotension can precipitate myocardial or renal ischemia in this group. By attenuating vasodilation and preserving SVR, remimazolam mitigates this risk. Our finding of 3 mmHg higher MAP with remimazolam, together with the literature, suggests fewer hypotensive episodes. In sum, remimazolam’s pharmacology (rapid-onset ester hydrolysis and limited direct myocardial suppression) produces a stabler blood pressure profile, which is particularly advantageous given the reduced cardiovascular reserve in older patients.

### 4.3. Safety Profile

Remimazolam’s overall safety profile compares favorably to propofol. Propofol’s potent respiratory depression and lack of antagonists make apnea and hypoventilation important risks. Remimazolam, like other benzodiazepines, causes less respiratory drive depression and can be reversed with flumazenil if needed [28,33]. In our meta-analysis, respiratory complications were numerically less frequent with remimazolam (RR = 0.49), echoing broader evidence: a large meta-analysis of procedural sedation found remimazolam dramatically reduced respiratory depression (RR = 0.40) and hypoxemia (RR = 0.34) versus propofol [33]. Bradycardia also appears reduced with remimazolam: multiple studies report fewer bradyarrhythmias under remimazolam, and our pooled HR was slightly higher with remimazolam. For instance, An et al. [33] reported remimazolam cut bradycardia risk by two-thirds (RR = 0.34) during sedation, and a recent elderly anesthesia trial observed significantly lower bradycardia (7.3% vs. 16.4%, *p* = 0.037) [31]. These data suggest remimazolam’s weaker vagotonic/sympatholytic effect leads to fewer heart-rate drops. Importantly, postoperative nausea and vomiting were no worse with remimazolam: our pooled RR was 0.93 (*p* = 0.86), and a focused meta-analysis found no postoperative nausea or vomiting difference vs. propofol [34]. Indeed, propofol has intrinsic antiemetic action, so parity implies remimazolam is at least non-inferior in this regard.

### 4.4. Recovery and Anesthesia Efficiency

In terms of recovery, remimazolam proved non-inferior to propofol. Our pooled analysis showed no significant difference in time to emergence or PACU length of stay. This indicates that remimazolam’s rapid ester hydrolysis translates into clinically similar recovery. In fact, remimazolam has a short context-sensitive half-life and can be reversed with flumazenil to expedite awakening [28]. Some individual studies even report faster waking. For example, one Chinese RCT found significantly shorter recovery and PACU times with remimazolam [24], and a broader meta-analysis by Luo and Tang [35] found remimazolam shortened time to consciousness by 5 min. In practice, these advantages were small and did not alter overall anesthesia workflow, as reflected by our MD of –1.5 min (*p* = 0.42) in recovery. Likewise, intraoperative remifentanil usage was identical between groups, indicating that analgesic requirements for colon surgery (and thus surgical stress) were unaffected by anesthetic choice. Anesthesia duration was unchanged as well, confirming that remimazolam does not delay case turnover. Notably, with respect to cognitive and delirium outcomes, existing evidence suggests no disadvantage to remimazolam use. Postoperative delirium is a major concern in elderly surgical patients, but meta-analyses have found comparable delirium rates between remimazolam and propofol, with remimazolam even reducing postoperative hypotension (a delirium risk factor) [36]. Wang et al. [24] found lower sedation-agitation and confusion scores with remimazolam.

### 4.5. Oncologic Surgical Context

Colorectal cancer surgery imposes unique anesthetic demands that underscore the importance of our findings. These operations are often lengthy (3–4 h), involve major visceral handling, and typically entail significant fluid shifts (from bowel preparation, irrigation, and bleeding) and fluctuations in sympathetic tone. Ensuring stable hemodynamics is critical: aggressive fluid resuscitation to counteract vasodilation can cause tissue edema, whereas untreated hypotension can compromise coronary and renal perfusion, especially in elderly or comorbid patients [32]. Furthermore, many colorectal procedures use pneumoperitoneum (laparoscopy), which elevates intra-abdominal pressure and afterload, challenging venous return. Under such stress, even small improvements in blood pressure control can translate to fewer organ insults. In this light, remimazolam’s ability to maintain MAP a few mmHg higher and reduce hypotension incidence is clinically meaningful. Indeed, Wang et al. [24] found that in colorectal cancer surgery, remimazolam outperformed propofol, yielding better MAP and fewer adverse reactions (10.6% vs. 37.3%).

Beyond hemodynamics, anesthetic choice may affect oncologic outcomes (through immune modulation), although evidence is still evolving. It is noteworthy that propofol-based anesthesia has been linked to improved cancer survival compared to volatile anesthesia in some studies, raising questions about how remimazolam might fit into oncologic anesthesia protocols. While our analysis did not address long-term cancer outcomes, the stable profile and low immunosuppressive burden of remimazolam are theoretically favorable. In sum, colon cancer resection demands an anesthetic that preserves perfusion and minimizes stress. Remimazolam’s pharmacodynamics with preserved vascular tone and rapid offset make it particularly well suited to this setting, supporting stable physiology throughout lengthy oncologic cases.

Based on the available evidence, remimazolam and propofol demonstrate broadly comparable anesthetic efficacy; however, certain clinical contexts may favor one agent over the other. Remimazolam may be preferable in elderly patients (>65 years), particularly those with reduced cardiovascular reserve, as well as in individuals with coronary artery disease, heart failure, or other conditions limiting cardiac compensatory capacity. It may also be advantageous in patients at increased risk of intraoperative hypotension, in frail individuals or those classified as ASA III–IV, and in situations where pharmacological reversibility is desirable given the availability of flumazenil. In these settings, even modest improvements in hemodynamic stability and potentially lower respiratory depression may offer clinical benefit, although robust outcome-driven data remain limited. Conversely, propofol may remain preferable in younger, hemodynamically stable patients, in clinical environments prioritizing extremely rapid induction and widespread familiarity, in institutions with cost constraints due to its lower acquisition cost and broad availability, and in centers with well-established ERAS pathways built upon propofol-based anesthetic protocols. Ultimately, anesthetic choice should be individualized according to patient characteristics, institutional practices, clinician expertise, and economic considerations, pending further high-quality trials powered for clinically meaningful endpoints.

### 4.6. Strengths and Limitations

This systematic review and meta-analysis has several strengths. We synthesized all available data specifically in colon cancer surgery, yielding a homogeneous patient population (all older adults undergoing elective colorectal resection with similar operative times). The trials were generally well matched at baseline, and we applied rigorous meta-analytic methods (random-effect models, and timepoint subgrouping) to derive robust estimates. However, limitations must be acknowledged. Only six trials were identified, with a total of 542 patients. This limited sample means some analyses (especially on rare adverse events) are underpowered. Several outcomes showed high heterogeneity (e.g., I^2^ > 75% for recovery times), reflecting differences in study protocols, anesthesia regimens, or measurement. All included studies were conducted in similar healthcare settings (predominantly Asian centers), potentially limiting generalizability to other populations or surgical styles. The trials also varied in specific definitions (e.g., for hypotension or respiratory events) and blinding, introducing bias risk. Our review focused on short-term intra- and postoperative endpoints. Data on long-term outcomes (e.g., postoperative cognitive dysfunction, cancer recurrence) were not available.

### 4.7. Recommendations

Clinically, our findings suggest remimazolam is a viable alternative to propofol for anesthesia in older colon cancer patients. Given its more stable hemodynamic profile, remimazolam should be strongly considered for patients at high risk of hypotension or myocardial ischemia (advanced age and cardiovascular comorbidities). Its similar recovery profile means no loss of efficiency in the operating room. In practice, anesthesiologists may incorporate remimazolam into multimodal anesthesia protocols (for example, combining it with regional blocks or non-opioid analgesics) to further minimize hemodynamic swings. Because remimazolam is reversible with flumazenil, teams should ensure availability of the antagonist for rapid emergence if needed. For future research, targeted trials are needed. Studies in very frail patients or those with ASA III–IV status could clarify whether the hemodynamic advantage translates into improved outcomes (fewer perioperative infarctions and acute kidney injuries). Investigations of remimazolam in the context of enhanced recovery protocols (ERAS) or opioid-sparing techniques would be valuable. Critically, the impact of remimazolam on neurocognitive outcomes (postoperative delirium and long-term cognitive function) and oncologic metrics (immune function and tumor recurrence) should be assessed in large, long-term RCTs. Such trials should also collect patient-centered outcomes (pain scores and satisfaction) and cost-effectiveness data, given the higher acquisition cost of new drugs. Overall, further high-quality trials will help define remimazolam’s role in optimizing anesthesia for complex cancer surgeries.

## 5. Conclusions

Remimazolam provides anesthesia for colon cancer surgery that is at least as effective as propofol, with small, but meaningful improvements in hemodynamic stability. Our meta-analysis found remimazolam was associated with higher intraoperative MAP and marginally higher HR, while recovery and remifentanil use were equivalent. Adverse events were no worse (and often better) with remimazolam: notably, there were trends toward fewer hypotension and respiratory events. These findings suggest that remimazolam may be a safer choice for elderly surgical patients, especially those at risk of cardiovascular compromise. As anesthetic practice evolves, remimazolam merits consideration in the anesthesiologist’s armamentarium for high-risk oncologic cases. Future research should build on this evidence base by examining long-term and population-specific outcomes, but current data support remimazolam’s clinical benefits in the colorectal surgery setting.

## Figures and Tables

**Table 1:** Baseline characteristics of the patients in the included studies.

Study ID	Study Design	Sample Size		Age, Mean (SD)		Male, *n* (%)		BMI, Mean (SD)		Surgery Duration, Mean (SD)		ASA Grade 1, *n* (%)		ASA Grade 2, *n* (%)	
		R	P	R	P	R	P	R	P	R	P	R	P	R	P
Liu 2024 [22]	RCT	50	50	71.620 (5.47)	71.400 (5.5)	22 (44)	21 (42)	23.109 (2.872)	23.953 (3.326)	212.7 (50.68)	200.8 (52.473)	27 (54)	29 (58)	18 (36)	15 (30)
Liu 2025 [23]	RCT	30	29	73.9 (6.3)	71.5 (5.2)	14 (46.6)	15 (51.7)	21.6 (1.97)	21.57 (2.13)	210 (33.6)	218.2 (37.4)	17 (56.6)	17 (58.6)	13 (43.3)	12 (41.4)
Tian 2025 [26]	RCT	38	40	66.79 (5.08)	65.65 (4.54)	26 (68.4)	24 (60)	23.65 (2.53)	24.23 (2.93)	26.66 (10.88)	26.13 (9.13)	NR	NR	NR	NR
Wang 2025 [24]	Cohort	85	75	58.01 (3.82)	57.76 (3.87)	43 (50.6)	37 (49.3)	22.49 (2.49)	23.09 (3)	188.12 (22.01)	190.16 (18.08)	40 (47.1)	32 (42.7)	45 (52.9)	43 (57.3)
Wu 2024 [25]	RCT	45	45	61.2 (10.3)	58.5 (9.9)	28 (62.2)	25 (55.5)	21.8 (2.9)	22.1 (3.3)	232.1 (70.5)	235.9 (73.8)	8 (17.7)	7 (17.02)	37 (82.2)	38 (84.4)
Xin 2022 [27]	RCT	28	27	54.11 (12.08)	56 (10.13)	20 (71.4)	17 (62.9)	22.2 (1.71)	22.28 (1.61)	NR	NR	23 (82.1)	20 (74.1)	5 (17.9)	7 (25.9)

R: remimazolam group; P: propofol group, SD: standard deviation, BMI: body mass index, RCT: randomized controlled trial, NR: not reported, ASA: American Society of Anesthesiologists.

**Table 2:** Quality assessment of the cohort study using NOS.

Study ID	Selection (Max. 4)	Comparability (Max. 2)	Outcome (Max. 3)	Total (Max. 9)
Ld Wang 2025 [24]	☆☆☆☆	☆☆	☆☆☆	☆☆☆☆☆☆☆☆☆

## Data Availability

No new data were created or analyzed in this study. Data sharing is not applicable to this article.

## Supplementary Materials

The following supporting information can be downloaded at: https://www.mdpi.com/article/10.3390/pharmaceutics18040448/s1, Table S1: PRISMA 2020 Checklist.
